# Treating Psychological Trauma in the Midst of COVID-19: The Role of Smartphone Apps

**DOI:** 10.3389/fpubh.2020.00402

**Published:** 2020-08-18

**Authors:** Jamie M. Marshall, Debra A. Dunstan, Warren Bartik

**Affiliations:** Faculty of Medicine and Health, School of Psychology, University of New England, Armidale, NSW, Australia

**Keywords:** smartphone apps, COVID-19, trauma, PTSD, mHealth, anxiety, Internet, mental health

## Abstract

With the COVID-19 pandemic confronting health systems worldwide, medical practitioners are treating a myriad of physical symptoms that have, sadly, killed many thousands of people. There are signs that the public is also experiencing psychological trauma as they attempt to navigate their way through the COVID-19 restrictions impinging on many aspects of society. With unprecedented demand for health professionals' time, people who are unable to access face-to-face assistance are turning to smartphone apps to help them deal with symptoms of trauma. However, the evidence for smartphone apps to treat trauma is limited, and clinicians need to be aware of the limitations and unresolved issues involved in using mental health apps.

## Introduction

Although many medical and allied health professionals are conducting telehealth sessions with patients and clients during the COVID-19 pandemic, the increased level of demand means that some people may not be able to access services in an adequate timeframe (1). In response, increasing numbers of sufferers are turning to digitized, automated options such as mobile applications (apps) (2). For people with symptoms of acute stress disorder, which if present for longer than a month is reclassified as posttraumatic stress disorder (PTSD) (3), mental health apps are potentially one way to access treatment and lessen the burden on primary health care.

It is no surprise that individuals are turning to digital options—over 5.2 billion people worldwide own a smartphone (4). When the COVID-19 situation rapidly worsened, downloads of mental health apps accelerated (2, 5). For example, the apps *Calm* and *Headspace* ranked two and three for worldwide revenue achieved in March 2020 for health and fitness apps in the Google Play store, achieving sales worth over US$1,149,000 and US$838,000, respectively (6). Around the world, there is other evidence that both authorities and people are turning to apps and other digital options in large numbers to cope with the trauma of COVID-19: in Australia (7), China (8), India (9), New Zealand (10), U.K. (11), U.S. (5), and others. However, we do not yet have accurate data on how many people are experiencing symptoms of PTSD as a result of COVID-19, nor how many are relying on smartphone apps to cope with these symptoms.

## The Evidence for Treating Symptoms of Trauma With an APP

Best practice involves initially treating symptoms of trauma with specially administered psychological therapy, such as trauma-focused cognitive-behavioral therapy (CBT), eye movement desensitization and reprocessing (EMDR), cognitive processing therapy, narrative exposure therapy, or prolonged exposure therapy (12). If there is no or little improvement in symptoms as a result of these treatments, clinicians may explore pharmacological options, either as an adjunct to therapy or as a front line treatment if psychotherapy has been ineffective (13). If psychotropic medication is prescribed for PTSD, it will usually initially be an antidepressant, and mostly it will be a selective serotonin reuptake inhibitor (SSRI) or serotonin-norepinephrine reuptake inhibitor (SNRI) (12). However, the evidence for efficacy of pharmacological treatments of PTSD is less than that for psychological treatments (13), so further treatment options would be welcomed, especially with the anticipated worldwide surge in trauma-related presentations that may arise during and after the COVID-19 situation with both health workers and the general public (14–17).

The description of “antidepressant” for a class of drug is confusing because many antidepressants not only treat depression, but also anxiety (18). When pharmacological treatment is prescribed for anxiety or depression in the form of an antidepressant, it is has been done so for a medication that has been approved by government regulators in that country that have identified the medication as safe and efficacious. A *digital antidepressant*, therefore, can be thought of as any app, website or other digital tool that is specifically designed to treat symptoms of anxiety or depression. If this digital tool is a form of non-pharmaceutical medication, there is a need for these digital tools to have research behind them. Government regulators would not allow a pharmaceutical antidepressant to become available without research of efficacy, and using the term “digital antidepressant” is a reminder of how important it is to consider apps that claim to treat anxiety and depression symptoms as things that need to have research for their efficacy as well. The idea that a health professional could “prescribe” a mental health app (19) is attractive at this time because of the difficulties accessing face-to-face treatment, whether by telehealth or in-person. Medical practitioners are concerned about the welfare of their patients, and if their patients cannot access “human” help, they may seek out other options in the form of digital technology. However, clinicians should be aware of the evidence for mental health apps, and understand that a digital antidepressant may not produce the desired clinical outcomes.

Similarly, the above mentioned evidence-based psychotherapies for treating symptoms of trauma require the specialized skills of highly trained practitioners. To be effective, such practitioners have to be experts in recognizing the signs and symptoms of trauma, adapting their psychotherapy in response to changes in client presentation, and acting appropriately if their client's condition deteriorates, especially in response to a risk of self-harm or suicide. If a general practitioner or other health professional does not have such specialized training in the area of psychologically treating symptoms of trauma, they may have the mistaken belief that a mechanized version of psychotherapy is possible in an app without being aware that evidence may not exist for the app's effectiveness in treating trauma presentations.

Evidence for the efficacy and effectiveness of mental health apps is limited (20, 21). The research suffers from methodological deterrents (e.g., quality randomized controlled trials [RCTs] can be expensive and take years to run which is an impediment for the profit-driven app sector), heterogeneity across studies, no published replication studies to speak of, and a lack of independence (i.e., studies completed by researchers who have not had any association with the app) (22, 23). This last point is important and is illustrated through a comparison with chemical antidepressants.

The early evidence base for chemical antidepressants, which was largely established by pharmaceutical companies who developed these medications, showed far higher effect sizes and greater levels of statistical significance than more recent studies (24). The later studies have included a much greater proportion of independent trials by researchers who have no association with the medications being tested. Their results have demonstrated significantly less efficacious and effective outcomes (24). Using the Cochrane Bias Tool, it has been estimated that 82% of all previous published studies on antidepressant medications are at moderate or high risk of bias due to the involvement of pharmaceutical companies (24).

If the efficacy of mental health apps is to be free of the limitations affecting many drug trials, unbiased research is required while their development is still relatively young. In a recent review of the two major app stores, only 1% of apps that claimed to offer a therapeutic treatment for anxiety and depression had independent research to back-up claims of efficacy (25). That is, research that was conducted by individuals, institutions or organizations who were not involved in the development of the app, and who would not stand to gain financially or otherwise from it. This is not to discourage research by app developers—quite the contrary. More app developers also need to conduct research on their product because a huge proportion of publicly available apps have no research support whatsoever (22, 23). While much of the research conducted by those who have an association with the app may be of acceptable quality, it is important that independent research in this area is increased to further legitimize the evidence base and minimize concerns regarding bias.

There is less evidence for apps specifically treating symptoms of trauma. The principal research on this front has come from the U.S. Department of Veterans' Affairs (VA), developers of *PTSD Coach* (26) and a suite of other apps, most of which are specifically aimed at veterans or their families. At time of writing, no published evidence could be found for the efficacy or effectiveness of any individual app specifically designed to treat symptoms of trauma, other than those produced by VA (27, 28). This is despite hundreds of publicly available apps purporting to do this (28, 29), and the availability of a standardized framework for developing PTSD-focused apps that stipulates the importance of demonstrated efficacy (30). Without a more diverse evidence base, it is unknown to what extent people benefit when they attempt to manage trauma by using apps. Worse still, we do not know how much damage is being caused by misinformed and poorly developed apps.

## Government Regulation

Examples of potential harm from an app include breaches of privacy, misuse of personal data, providing inappropriate advice (31), and poor app functionality leading to possible app failure at a critical emotional point for the user. It has also been found that for over 20% of publicly available apps claiming to treat symptoms of PTSD, their app store descriptions did not contain any specific PTSD evidence-based content (28). This is where government intervention is urgently required, but many governments are struggling to develop suitable regulations around mental health apps (32). Authorities such as the U.S. Food and Drug Administration are focused on removing apps that may cause harm (33). A recent report by the Australian Commission on Safety and Quality in Health Care (34) attempts to encompass a wider regulatory view of digital mental health resources founded on a “model of care” with “best available evidence and best practice” (p. 20).

However, these new standards in digital mental health care, which include mental health apps, are yet to be implemented. In the meantime, medical practitioners who are expecting an influx of trauma-related presentations in the wake of COVID-19, must use caution in directing patients toward apps to assist in managing symptoms of trauma.

## Discussion

There is uncertainty about the effectiveness of many mental health apps (i.e., their ability to deliver beneficial treatments in a real world setting) and the most appropriate methods of examining this (35). The COVID-19 crisis brings to the fore the need for a centralized database of information for use by medical professionals, governments, therapists, researchers, and consumers (36). Clearly, there is a need to move beyond reliance on app store ratings and reviews, which may be inaccurate, ill-informed, or fake (37), to the requirement for app researchers to provide accessible and timely research evidence. While the time demands of the gold standard RCT can be an impediment to research, other methodologies that do not sacrifice scientific rigor and integrity can potentially be conducted on apps in a more timely manner (38). These include scalable single-case designs involving practicing clinicians working with researchers (36). In such a model, clinicians could contribute their findings to a centralized database that may continually be updated with results that occur from individuals using their app in real-world settings. The single-case methodology has the added advantage of being able to provide potentially more information on the characteristics of the individual than might otherwise be identified in larger RCT designs. This may in-turn lead to more informed hypotheses about how individual characteristics may impact on the effectiveness of a mental health app.

Other than the need for further research on efficacy and effectiveness, future development of apps for treating symptoms of trauma (and indeed for all apps treating mental illness generally) needs to take into account a number of factors, and there are many existing development blueprints that app developers can refer to (39–41). Building a mental health app on the foundations of an evidence-based framework is vital (42). There are several evidence-based frameworks that inform PTSD psychological treatments (as mentioned above), and it would seem plausible that such treatment interventions could be incorporated into an app. There needs to be expert input from qualified clinicians and/or researchers into the development of a mental health app—many apps claiming to treat symptoms of mental illness do not have such input (25, 43, 44). Given that the development of mobile mental health apps is still in its infancy, we are still not certain about the mechanisms of action of such apps, and therefore the level of importance of characteristics such as app design and usability is still being investigated. However, it would seem plausible to assume that a mental health app has to be easy to use, engaging, and aesthetically pleasing to be efficacious and effective (41), and therefore having the input from experienced app designers would seem to be a necessity. This is only a brief summary of necessary aspects of a successful mental health app, but even here it can be seen that many different aspects of development need to be considered before an app is able to make claims that it can successfully treat symptoms of trauma, or of any other mental illness.

Another concerning feature of around 40% of mental health apps is that they lack a publicly accessible privacy policy (45). Given the sensitive nature of people's mental health information, app developers need to pay more attention to this. One reason why app developers have been able to get away with this for so long is that there is no government regulation about the need for privacy policies for digital mental health resources. If government authorities can broaden and strengthen their oversight of this sector, protecting people's privacy will be as necessary a factor as ensuring that mental health apps do no harm to their users.

There are websites that clinicians (and consumers) can access for reviews and further information about choosing mental health apps, listed in Table 1. The rationale for providing the information in Table 1 is so that clinicians can become more informed about recommending appropriate apps for their patients and clients. We did not recommend specific apps, as that is not the purpose of this paper. Although lacking information on how to measure effectiveness, these websites are nevertheless useful resources for clinicians who need assistance identifying potentially suitable apps. While the current evidence base is lacking, it is hoped the COVID-19 crisis is a potential catalyst for ensuring that mental health apps have demonstrated effectiveness for treating specified mental health disorders, including PTSD-related trauma, into the future.

**Table 1:** Website resources for clinicians that provide reviews and further information on mental health apps for symptoms of trauma.

**Resource**	**Summary**
*American Psychiatric Association's App Evaluation Model* (https://www.psychiatry.org/psychiatrists/practice/mental-health-apps/app-evaluation-model)	U.S.-based framework for clinicians and researchers on how to conduct their own evaluation of apps
*Anxiety and Depression Association of America* (https://adaa.org/finding-help/mobile-apps)	U.S.-based non-government, non-profit organization providing reviews of mental health apps by volunteers with recognized mental health qualifications who do not have any association with the apps being rated
*Beacon* (https://beacon.anu.edu.au/)	Australian university website providing research summaries of digital health resources, including mental health apps
*Head To Health* (https://headtohealth.gov.au/)	Australian government website providing information regarding digital mental health resources, including apps
*Health Navigator* (https://www.healthnavigator.org.nz/)	New Zealand government-supported website, with input from professional health-related bodies, providing information regarding digital health and medical resources, including mental health apps
*mHabitat* (https://wearemhabitat.com/)	U.K. government-supported website, with the involvement of various departments of the National Health Service, dedicated to developing partnerships with developers of digital health solutions, including mental health apps
*MindApps* (https://mindapps.dk)	A mental health app review website by The Centre for Telepsychiatry, Psychiatry in the Region of Southern Denmark. It includes reviews by therapists, academics, and consumers
*NHS Apps Library* (https://www.nhs.uk/apps-library/)	Coordinated by the U.K.'s National Health Service, this tool allows users to search for all types of health apps, including mental health apps, with summaries about what the app does, links to the app's website, and links to the App Store and/or Google Play for download.
*The Organization for the Review of Care and Health Apps (ORCHA)* (https://www.orcha.co.uk/who-we-help/health-and-care/)	Private organization based in the U.K. offering a number of tech-related health services, including reviews, accreditation, curation and prescription services for health and mental health apps
*Reachout.com* (https://au.reachout.com/tools-and-apps)	Australian non-government organization providing expert and consumer reviews on mental health apps
*PsyberGuide* (https://psyberguide.org/)	U.S. non-government organization providing expert reviews on mental health apps

## Data Availability Statement

All datasets analyzed in this study are included in the article/supplementary material.

## Author Contributions

JM wrote the manuscript drafts. DD and WB provided proofreading and editing. All authors read and approved the final submitted version.

## Conflict of Interest

The authors declare that the research was conducted in the absence of any commercial or financial relationships that could be construed as a potential conflict of interest.
